# Skin stretch suturing with Nice knots in the treatment of small- or medium-sized wounds

**DOI:** 10.1186/s13018-020-02007-8

**Published:** 2020-10-22

**Authors:** Jianmin Xu, Rui Chang, Wei Zhang, Chengcheng Zhang, Dezhi Zhu, Fanxiao Liu, Yongliang Yang

**Affiliations:** 1 Department of Orthopaedics, Juye People’s Hospital, Heze, Shandong China; 2 Yudong Gu’s Academician Workstation, Heze Boai External Microscopic Orthopedic Hospital, Heze, Shandong China; 3 Department of Orthopedics, Shandong Provincial Hospital Affiliated to Shandong University, No. 324, Road Jing Wu Wei Qi, Jinan, 250021 Shandong China; 4 Department of Orthopedics, Heze Peony People’s Hospital, Heze, Shandong China; 5 Department of Orthopedics, Shandong Provincial Hospital Affiliated to Shandong First Medical University, 250021, Jinan, Shandong China

**Keywords:** Wounds, Closure, Soft-tissue defects, Suture, Nice knot

## Abstract

**Background:**

To investigate the clinical efficacy and outcomes of skin stretch suturing with self-locking sliding Nice knots in the treatment of small- or medium-sized wounds.

**Methods:**

From June 2015 to May 2018, 26 patients with small- or medium-sized wounds were included in the present study. Skin stretch suturing with self-locking slide Nice knots was performed to gradually close the soft-tissue defects in these patients. The time of wound closure and healing was recorded. The color and blood supply of the skin, cutaneous sensation, the stretch of skin, and the hair growth situation of the skin wound were observed and recorded.

**Results:**

There were 17 males and 9 females with an average age of 30.65 years (range, 15–48 years). The areas of the soft-tissue defects were between 3.2 × 7.1 cm and 8.0 × 15.2 cm. All patients underwent stretch suturing with self-locking slide Nice knots to close the soft-tissue defects. All wounds were successfully closed and healed. The mean time of wound closure was 10.69 days (range, 5–20 days), and the mean time of wound healing was 16.85 days (range, 10–24 days). The cutaneous sensation of skin wound recovered normally, and the color of the skin wounds was the same as that of normal skin at the last follow-up. The hair growth situation of the skin wounds also returned to normal.

**Conclusions:**

This study revealed that Nice knots yielded an accepted clinical result as a new method to close small- or medium-sized wounds that was simple and less minimally invasive, resulted in progressive tension, did not return to previous results, and partially replace flaps or free skin grafts.

## Introduction

Wounds with small- or medium-sized soft-tissue defects are a common problem after soft tissue injuries and scar resection and are mostly difficult to suture directly after debridement. Numerous traditional methods are used to repair this type of wound with these small- or medium-sized soft-tissue defects, including skin grafts, focal perforator flaps, free skin and soft tissue flaps, and skin expanders or stretchers [1, 2]. However, these traditional methods are limited in their use because of the damage to the donor site, massive blood loss, complex procedures, long learning curve, and heavy economic burden for patients. The conventionally used surgical sutures and knots are sufficiently strong, but are not gradually tightened to close the wound. Several operations are needed to close the wounds; therefore, the economic burden on patients is increased, and some patients have extreme difficulty tolerating the associated pain. It would be beneficial for patients with soft-tissue defects if the wound could be gradually closed with a simple procedure.

Nice knots represent a new type of self-locking sliding knot; they were first reported by Dr. Pascal Boileau [3] in the Nice area of France in 2017 and are usually used in the arthroscopic repair of rotator cuff tears and tuberosity fixation during hemiarthroplasty for proximal humerus fracture. There are different from the square or simple knots. The latter two types of knots are the most common knots used in surgical procedures. The square knot is used for various suture methods to prevent slipping due to double-knot sutures, and the simple knot can be tightened but easily loosens. The Nice knot, a double-stranded knot with double the suture strength, is easy to perform and does not loosen after tightening. The doubling of the suture also results in increased internal friction, which translates into excellent loop and knot security [4]. The Nice knot can be applied in both open and arthroscopic surgery to fix torn tendons/ligaments or fractured/osteotomized bones [3, 5]. To our knowledge, there have been no reports on the application of Nice knots for the closure of wounds with small- or medium-sized soft-tissue defects.

The purpose of this study was to assess the effectiveness and clinical outcome of skin stretch suturing with self-locking sliding Nice knots in the gradual closure of wounds with small- or medium-sized soft-tissue defects that cannot be closed directly. The working hypothesis is that sutures with the self-locking sliding Nice knots have the beneficial effect of convenience in the closure of wounds with small- or medium-sized soft-tissue defects.

## Materials and methods

This retrospective study was approved by the ethics committee of our institution. The inclusion criteria were patients with small- or medium-sized soft-tissue defects after soft-tissue injuries or tension-reduced incisions or ulectomy; the wound could not be closed directly. The exclusion criteria were massive soft-tissue defects, infectious wounds, wounds with serious bone and tendon injuries, and severe medical comorbidities or an inability to comply with postoperative cooperation to tighten the Nice knots for wound closure.

From June 2015 to May 2018, 26 patients with small- or medium-sized soft-tissue defects were treated by skin stretch suturing with Nice knots in our hospital. Seventeen patients were male, and 9 patients were female. The mean age of these patients was 30.65 ± 8.64 years (range, 15–48 years). The types of soft-tissue defects were traumatic in 9 patients, tension reduced incisions in 7 patients, skin defects and tendon exposures in 5 patients, skin and soft-tissue defects after ulectomy in 3 patients, and skin defects at their donor site in 2 patients. There were 18 cases of lower-extremity defects, 6 cases of upper-extremity defects, and 2 cases of defects at other sites. The sizes of the soft-tissue defects were between 3.2 × 7.1 cm and 8.0 × 15.2 cm (Table 1). The definition of the wound criteria followed these references [6–8].

**Table 1:** The demographic data of the included patients. M, male; F, female

No. of patient	Gender	Age, yrs	Cause of soft-tissue defects	Site	Sizes (cm)	Time of wound closure (days)	Time of woundhealing (days)	Follow-up (months)
1	M	26	traumatic	Forearm L	4.2 × 8.5	8	14	6
2	F	32	tendon exposures	Calf R	5.6 × 12.1	1**2**	20	9
3	M	15	traumatic	Calf L	4.0 × 9.6	11	18	6
4	F	41	after ulectomy	Thigh L	6.5 × 13.4	6	15	8
5	M	36	tension reduced incision	Forearm R	3.2 × 7.1	5	14	12
6	M	23	traumatic	Calf R	7.0 × 13.8	16	21	8
7	M	45	tension reduced incision	Abdomen	4.5 × 9.0	8	10	9
8	F	30	traumatic	Calf L	4.6 × 11.3	10	18	12
9	M	43	tendon exposures	Foot L	4.1 × 11.2	13	20	8
10	M	28	tension reduced incision	Calf R	6.4 × 13.6	18	24	9
11	F	39	traumatic	Thigh R	5.3 × 14.1	9	14	6
12	M	34	tension reduced incision	Calf L	8.0 × 15.2	13	24	12
13	M	19	after ulectomy	Upper arm R	5.0 × 13.4	10	16	8
14	F	23	traumatic	Thigh R	6.1 × 14.5	7	14	9
15	M	21	tendon exposures	Calf R	5.3 × 13.8	9	18	6
16	M	36	tension reduced incision	Forearm L	6.4 × 13.7	11	15	6
17	M	31	donor site	Calf R	5.8 × 13.4	12	14	6
18	F	48	traumatic	Forearm L	5.1 × 12.9	9	15	9
19	M	34	tendon exposures	Calf L	4.6 × 10.4	20	21	12
20	M	38	tension reduced incision	Thigh L	4.2 × 11.3	8	14	6
21	F	27	tendon exposures	Calf R	3.8 × 8.6	13	18	6
22	F	24	donor site	Calf L	4.5 × 11.6	16	21	9
23	M	18	traumatic	Abdomen	5.2 × 12.4	7	10	9
24	M	36	after ulectomy	Calf R	5.3 × 10.7	10	16	12
25	M	42	traumatic	Calf R	7.5 × 3.2	5	16	6
26	F	25	tension reduced incision	Upper arm R	4.3 × 12.5	12	18	9
		30.65 ± 8.64				10.69 ± 3.81	16.85 ± 3.66	8.39 ± 2.19

### Surgical procedure

#### General steps

According to the positions and areas of the soft-tissue defects and the patients’ tolerance, the appropriate anesthesia method was applied. The different positions of the patients were dependent on the site of the soft-tissue defects. The wound was thoroughly debrided first; the skin margin was dissociated, and the stretching direction was determined on the basis of the loosest part by a skin crushing test. After surgery, patient was given an intravenous drip of 10 mg of *Sodium Aescinate for Injection* (Luye corpation, Shandong, China) per day for a course of 7–10 days. We have provided information about medicine and their dosage and course that was given to reduce swelling.

#### Nice knots only

**Fig. 1:**
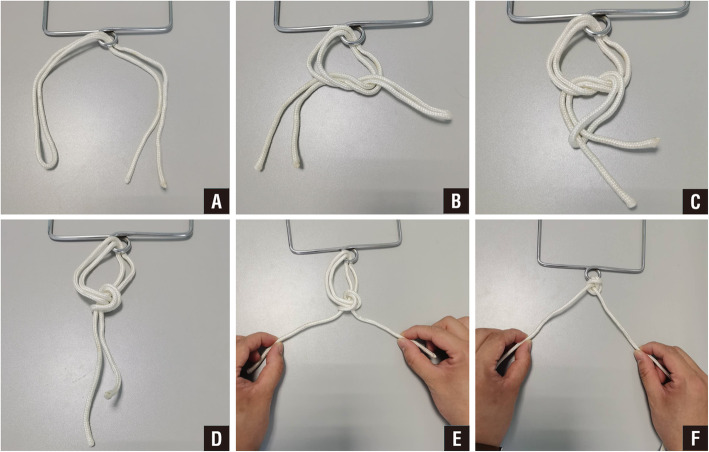
Nice Knot technique. **a** A doubled-over suture is passed around the tissue. **b** A single square knot is thrown. **c** The 2 free limbs are passed through the loop. **d** The knot is dressed. **e** The knot is slid down by pulling the 2 free limbs apart. The 2 limbs can also be pulled back toward the surgeon at once or alternately. A knot pusher can also be used to help the knot down while the limbs are being pulled. **f** The tightened knot is ready to be secured with 3 alternating half-hitches or surgeon’s knots

**Fig. 2:**
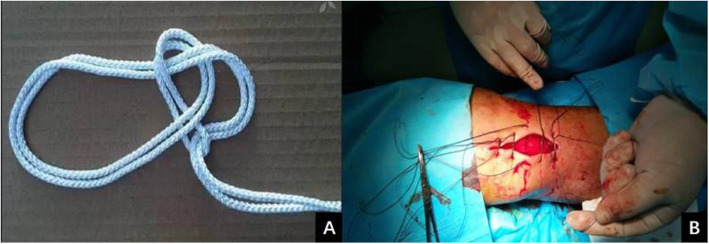
**a** Illustration of Nice knot. **b** The application of Nice knots for wound closure

The No. 1 MERSILK nonabsorbable sutures (ETHICON, Johnson & Johnson, USA) were folded into double strands and penetrated into the skin needle. The skin margin was interruptedly sutured with an edge distance of 1–2 cm and a needle distance of 1–1.5 cm. Several more sutures were added if the wound was large or irregular. The suture was passed around the tissues to be fixed and tightened with the Nice knot. The suture was doubled over itself to obtain 2 free limbs on one end and a loop on the other. A simple square knot was thrown using the loop on 1 hand and the 2 free limbs on the other. The loop was opened, and both free limbs were passed through it. The knot was then dressed by marking the smaller loop. When ready to secure the involved tissues, the surgeons tightened the sliding knot by pulling the 2 free limbs apart. There are many studies have introduced the Nice knot [3–5, 9]. We have presented a series of photo to show a step-by-step instruction about the Nice knot (Figs. 1 and 2). Then, the 2 free limbs were tightened for 30 s and relaxed circularly 1–2 min. The two skin edges were close to each other. Vaseline gauze and an aseptic dressing were used to cover the wound surface.

#### Nice knots + two parallel 1.5 mm K-wires

If the defect was large or the tension of the skin was too large for suturing, two parallel 1.5 mm K-wires were penetrated into the dermis layers of the two skin edges along with the longer axis of the wound, which the distance was 1.5 cm from the skin edges. Parallel 1.5 mm K-wires were penetrated into the dermis layers is not a new technique. Numerous studies [10–12] have been published for this technique. The sutures were outside of the K-wires and gradually tightened with the Nice knots. The spot stress was converted into linear stress to avoid cutting the skin margin on the basis of this method. If the muscles in the wound were swollen and blocked the skin from being closed, a 1 ml or 5 ml injection syringe was chosen for placement on the swollen muscles below the suture line to avoid cutting the muscles, and the Nice knot was gradually tightened to close the soft-tissue defects.

#### The tightening process

The affected limb was elevated to maintain warmth with a hot lamp. Medicine was used to improve the microcirculation of the affected limb. The color and circulation of the stretched skin edge was observed twice a day. The Nice knot was gradually tightened if there was no damage to the skin blood supply once every two days; otherwise, the process was postponed if the blood supply was affected. The knots were tightened again only after the blood supply recovered. The color and blood supply of the skin, cutaneous sensation, stretch of the skin, time of skin closure and healing, and hair growth situation of the skin wound were observed and recorded.

### Statistical analysis

All patients with small- or medium-sized soft-tissue defects were treated by skin stretch suturing with Nice knots. Sex distribution, and the sizes, types, and portion of soft-tissue defects were provided. The size of soft-tissue defects was presented in detail. Parametric data, including age, time of wound closure, and time of wound healing are described as the mean ± SD.

## Results

**Fig. 3:**
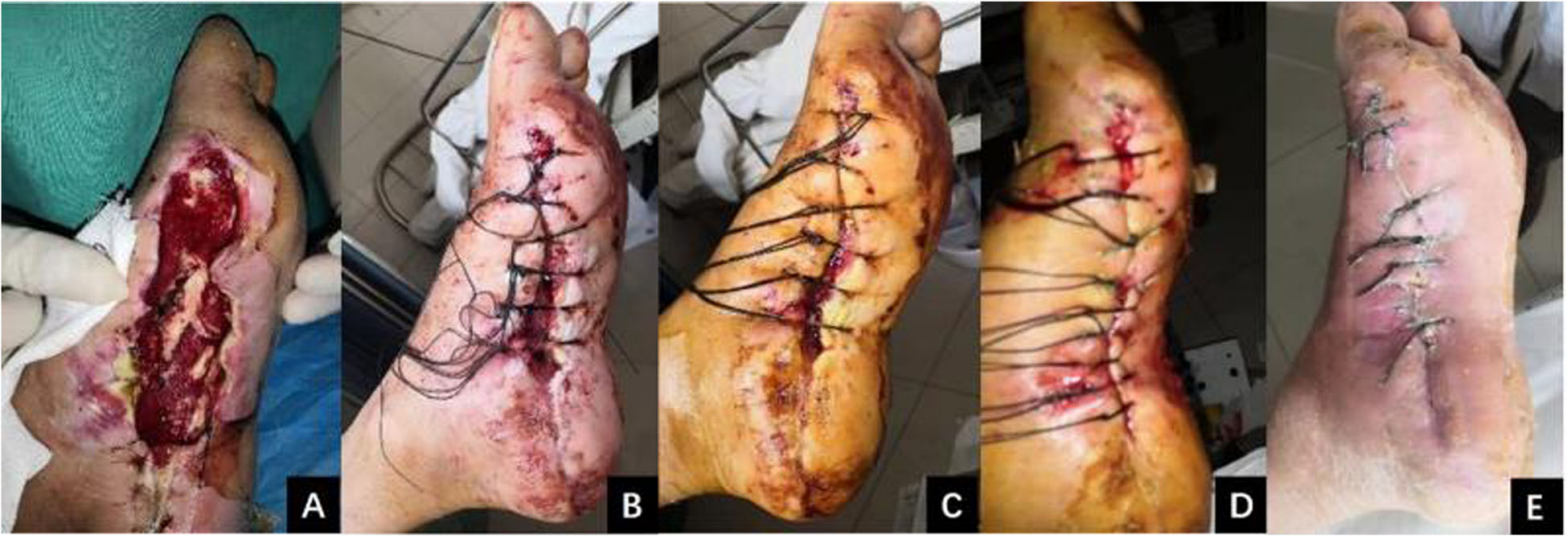
**a** Male, 43 years old, left foot with a degloved injury. A medium-sized soft-tissue defect (4.1 × 11.2 cm) was found on the medial side of the left foot after several debridement operations. **b**, **c** The technique of self-locking sliding Nice knots was used for the gradual stretch suturing of the defects. **d**, **e** The wound was closed after 13 days and completely healed after another 20 days

**Fig. 4:**
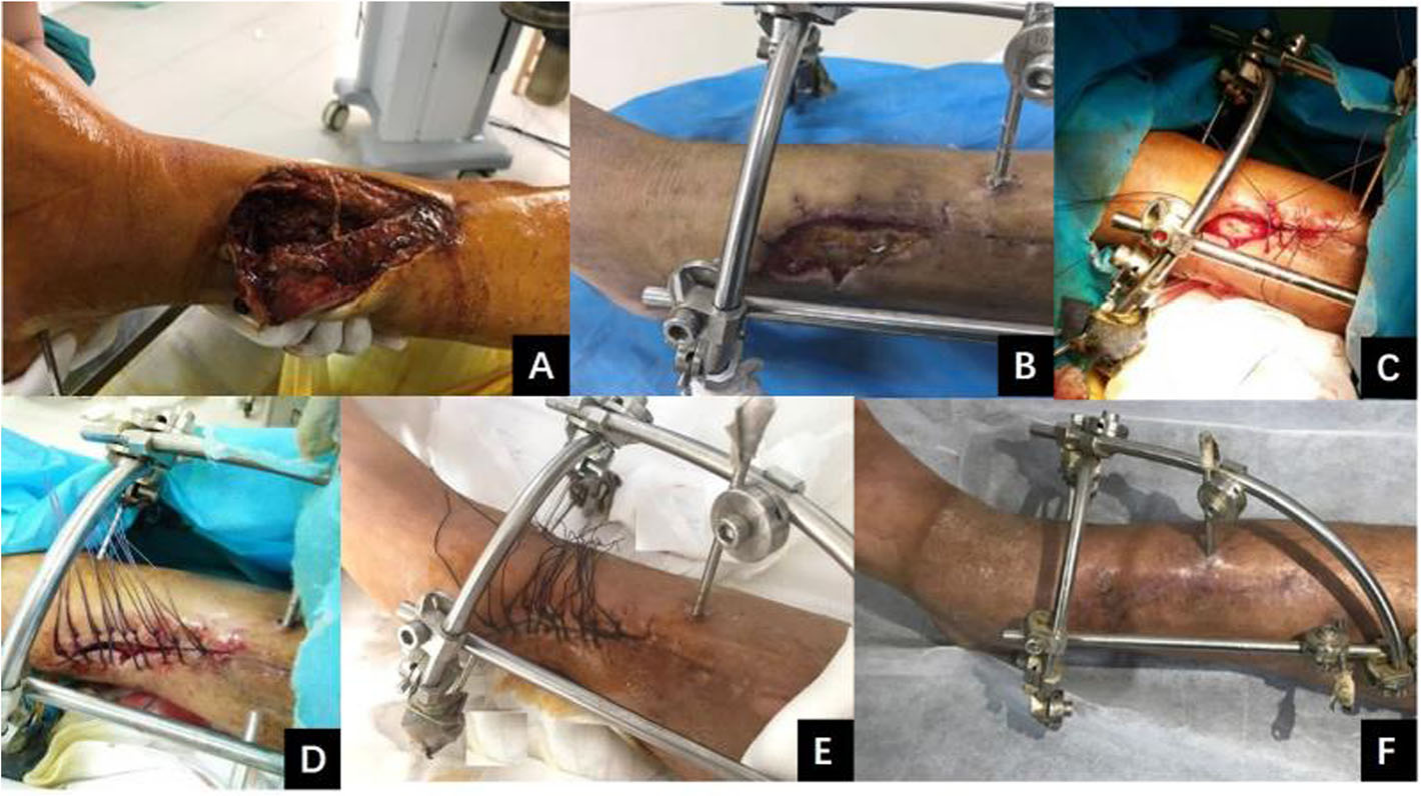
**a** Male, 42 years old, right tibia with an open fracture. The patient underwent emergency debridement, open reduction and hybrid external fixation. **b** The anterior tibial skin was necrotic (7.5 × 3.2 cm) and the bone was exposed 20 days later. **c**, **d** The technique of self-locking sliding Nice knots was used for gradual stretch suturing of the defect. **e** The wound was closed after 5 days. **f** The wound healed well when the patient returned for review 6 months after the operation

All patients with different degrees of soft-tissue defects underwent stretch suturing with self-locking sliding Nice knots (Figs. 3 and 4). All wounds were successfully closed and healed in this study. The mean time of wound closure was 10.69 ± 3.81 days (range, 5–20 days), and the mean time of wound healing was 16.85 ± 3.66 days (range, 10–24 days). A tension vesicle occurred in one case due to high skin tension 2 days postoperatively, and stretching restarted after the tension vesicle disappeared. No wound infection or skin necrosis was found in this study, and no patient underwent reoperation after stretch suturing with self-locking sliding Nice knots. The mean follow-up was 8.39 ± 2.19 months (range, 6–12 months). The cutaneous sensation of the skin wound recovered normally, and the color of skin wound was the same as that of normal skin at the last follow-up. There was no swelling or serious scar hyperplasia of the skin wound; meanwhile, the hair grew well, and the appearance was satisfactory.

Traditional methods for the closure of medium-sized soft-tissue defects are limited in their use because of the damage to the donor site, massive blood loss, complex procedures, long learning curve, and heavy economic burden for patients. During the continuous wound closure, we have included satisfaction survey that two patients have a bit of pain but it is tolerable. All patients satisfied with treatment because this simple procedure could decrease economic burden and has no complex surgery.

## Discussion

Soft-tissue defects are usually repaired by skin grafting [13], transferred free flaps [14], and skin expansion [15]. There are some problems with skin-grafting or free flap techniques [16], such as donor site damage [17], abnormal sensations [18], poor wear resistance of the grafted skin, bloated flaps [17], long learning curves for free or perforated flaps [19], and necrosis risks of the flap [20]. Skin soft-tissue expansion is a common surgical procedure to grow extra skin through controlled mechanical overstretching, which has been extensively applied in tissue repair and reconstruction in the field of plastic surgery for more than 30 years [15]. Skin expansion can be divided into internal expansion and external expansion. The aim of internal expansion is to detach the subcutaneous tissue and implant a dilator, resulting in the division of skin and other cells, and then “additional” skin can be acquired to repair the wound [21]. External expansion, which is also known as the skin stretching technique [10], pulls the normal skin on both sides of the wound edges to the center through external force and creates “additional” skin by linear loading with the help of skin viscoelasticity and extensibility to close wounds that are difficult to close conventionally [22]. Skin closure can be achieved with a skin dilator or a stretching device but is accompanied by complicated procedures and a high economic burden. The skin stretching device is essentially rigid; thus, it may produce unknowable tension in local tissue and usually causes necrosis, damage or avulsion of marginal wound tissue [10]. These methods are suitable for massive wounds with soft-tissue defects. For small- or medium-sized soft-tissue defects, skin stretch sutures with self-locking sliding Nice knots were first applied in this study. All wounds were gradually closed and successfully healed.

The characteristics of the skin are viscoelasticity, creep extensibility, and biological growth [23]. The viscoelasticity of skin is manifested in stress relaxation, which means that the intercellular spaces in the local skin tissues are opened and widened, while the skin tissue nearby shifts to a stretched area under pulling force [24]. The creep extensibility of skin is characterized by the rearrangement of collagen and elastic fibers after rapid expansion, and sometimes, type III collagen fibers and elastic fibers might rupture; therefore the skin is gradually elongated beyond its natural elongation and unable to ultimately return to its original position [25]. The skin can regenerate along the tension direction under the stimulation of stretching according to the tension-stress principle [26, 27]. On the basis of the viscoelasticity, creep extensibility, and biological growth of skin, the skin defects could be gradually closed with self-locking slide Nice knots in this study, a technique that was characterized by the simple procedure, strong tension, and gradual tightening [3]. The Nice knots could be gradually tightened once 2 days after the operation until the wound was closed. The mean time of closure of the soft-tissue defects was 10.69 ± 3.81 days without the need for more surgeries in this study.

However, to our knowledge, there have been no published studies on the effectiveness of Nice knots for soft-tissue defects. Our study was the first report about the application of Nice knots for wounds with soft-tissue defects. In our opinions, Nice knots can be used to close wounds caused by trauma, inflammation, tumor resection, bedsores, scar ulectomy, and the excision of skin grafts and flap donor sites. The contraindications of this method were patients with severe malnutrition, severe coagulation dysfunction, wounds without abundant blood supply, infectious wounds, and no insufficient normal skin around the defect. Nice knots were not used in areas of skin defects larger than 8.0 × 15.2 cm in this study. In the future, we will evaluate the clinical effects of applying Nice knots to massive wounds (larger than 8.0 × 15.2 cm).

The advantages of Nice Knot for soft-tissue defects were the simple procedure with few complications, the avoidance of damages to the donor sites, and the fact that skin maintained sensation and had more wear-resistance than flaps or grafted skin. The cost of this method was lower than that of skin expansion or skin stretching. The Nice knots could be applied to irregular wound areas. The disadvantages of this method are that it is not suitable for massive soft-tissue defects, there is pain when stretching the knots, and some tension scars formed after wound healing.

In our opinions, more attention must be paid to the following aspects. First, the Nice knots should be gradually tightened once every 2 days when swelling disappears and the blood supply. Second, Nice knots were not suitable for infectious or suspected infectious wounds; thorough debridement was necessary, and vacuum sealing drainage (VSD) was applied. Nice knots were applied to close the wounds after the granulation tissue in the wound was fresh. The closure speed was determined according to the blood supply of the skin and the patient’s pain tolerance. Finally, the removal time of the sutures should be prolonged to reduce tension scar formation.

There were some limitations in this study. First, this study was a retrospective clinical case analysis without a control group and was not a prospective study. Although we know that an included control group will make the study much stronger, it will be difficult for the close of size-mediate wound because of heterogeneous treatments. This study is just a new attempt that introduces a knotting method used widely for repairing rotator cuff tears in shoulder arthroscopy to close the skin wounds. In future, a randomized controlled trial will be performed to compare this new method with traditional methods for the closure of small- or medium-sized skin defects. A prospective study will be designed and applied in the future. Second, the sample size was not large enough because this was the first application of Nice knots on wounds with small- or medium-sized soft-tissue defects. Last, our clinical results are not being accurate because our follow-up time was not long enough.

## Conclusions

This study revealed that Nice knots yielded an accepted clinical result as a new method to close small- or medium-sized wounds that was simple and minimally invasive, resulted in progressive tension, did not return to previous results, and partially replaced flaps or free skin grafts.

## Data Availability

We do not wish to share our data, because some of the patient’s data regarding individual privacy, and according to the policy of our hospital, the data could not be shared with others without permission.

## Abbreviation

VSD: Vacuum sealing drainage
